# Mr. Edwards, on Umbilical Hernia

**Published:** 1808-11

**Authors:** G. F. Edwards

**Affiliations:** Member of the Royal College of Surgeons. Walcot Parade, Bath


### *To the Editors of the Medical and Physical Journal.*

GENTLEMEN,

IN your last month's Journal some remarks are made upon Exomphalos, or Umbilical Hernia, in which your correspondent, Mr. Bush, considers himself as the projector of an advantageous and new plan of cure in the infantile species of that disease; and also conceives, to use his own words, "that it is taken for granted, that surgeons are agreed, that umbilical hernia scarcely admits of radical cure in the adult." Does Mr. Bush suppose that it is a disease beyond the reach of human art finally to cure, merely because the patient, whose unhappy lot it should be to fall on, had reached the adult state?

Umbilical Hernia, it is true, in general, is not of that alarming nature; I say, in general, because the majority are composed of omentum only; but when it is formed of a portion of intestine, or both, and becomes strangulated, how should the surgeon act, if the protruded parts are not returnable? Surely, if reduction of the contents of this hernial sac could not be effected, the knife in the hands

 of of the skilful and well informed, would be the only resort, in order to save their patient; and this may be done with equal certainty and safety in strangulated umbilical hernia, as in any other form of that complaint.

It is, I presume, perfectly unnecessary to describe the usual modern practice of operating for umbilical hernia; but I would remark, that when the sac is sufficiently dilated, if the protruded parts are found in a fit state for reduction, and cannot be effected but by increasing the size of the opening into the cavity of the abdomen, it may be done without hazard in any direction, or the dread of dangerous peritoneal inflammation; but if the operator is fearful of wounding the ligament formed by the umbilical vessels, he may at all times avoid them, by making the incision upwards and outwards, on the left of the umbilicus; however, I am of opinion, from practical experience, that it is matter of little importance if the ligament should be divided. Many respectable modern authors I could quote to confirm this opinion.

The mode of radical cure for umbilical hernia, adverted to by the ancients; such as Guido, Albucasis, &c. was such, it must be admitted by all regular practitioners, as was at once extremely dangerous, excessively painful, and ineffectual to the proposed end, because they did not prevent a return of the disease; on the contrary, rendered every subsequent protrusion more alarming. The opinions and practice of such men as the above, but not of the "generality of Surgeons," might lead to the belief, that umbilical hernia in the adult scarcely admitted a radical cure.

Mr. Bush's mode of cure in the exomphalos of infants by pressure, is not by any means new, as the very plaster he describes was constantly used by my late worthy master Joseph Frowd, Esq. an eminent surgeon of Fremor, with the exception of the bandage of sticking plaster. The same plan I have for ten years invariably continued (securing the compress with a roller bandage) in numberless cases, and have found them individually succeed.

I beg it may be understood by your correspondent, Mr. Bush, that I have not any wish to arraign his chirurgical abilities, or his want of zeal in the profession; but I cannot conceive him well founded in his remark that exomphalos in the adult, "scarcely admits of radical cure," or in his reason, "that it is well known the danger which may may ensue by exciting inflammation in any of the cavities with in the abdomen."

It has fallen to my lot, though a young practitioner, to operate more than once or twice in this city, for several of the varieties of hernia, particularly femoral, with such complete success, as perhaps has given that confidence of which practical surgery alone is capable. It is not many months since I performed the operation for strangulated umbilical hernia in a female near fifty years old, with the happiest effect. In this case a portion of omentum, with the intestine, protruded, and formed a considerable tumor; every means of reduction had been tried, and all the horrible symptoms of the disease rapidly succeeding each other, the operation was considered the only resort; it was proposed to my patient with all that delicacy, sympathy, and feeling, which was demanded under circumstances so distressing; she immediately consented, and the event terminated in her perfect recovery.

I am, &c.

G. F. EDWARDS,

Member of the Royal College of Surgeons,

*Walcot Parade, Bath,*
*Oct.* 12, 1808.

